# Identifying Patients at Increased Risk for Poor Outcomes Among Poor-Grade Aneurysmal Subarachnoid Hemorrhage Patients: The IPOGRO Risk Model

**DOI:** 10.3390/jpm14111070

**Published:** 2024-10-24

**Authors:** Rustici Arianna, Scibilia Antonino, Linari Marta, Zoli Matteo, Zenesini Corrado, Belotti Laura Maria Beatrice, Sturiale Carmelo, Conti Alfredo, Aspide Raffaele, Castioni Carlo Alberto, Mazzatenta Diego, Princiotta Ciro, Dall’Olio Massimo, Bortolotti Carlo, Cirillo Luigi

**Affiliations:** 1 IRCCS Istituto delle Scienze Neurologiche di Bologna, UOSI Neuroradiologia Ospedale Maggiore, 40133 Bologna, Italy; arianna.rustici@ausl.bologna.it; 2 IRCCS Istituto delle Scienze Neurologiche di Bologna, UOC Neurochirurgia, 40139 Bologna, Italy; antonino.scibilia@ausl.bologna.it (S.A.); carmelo.sturiale@ausl.bologna.it (S.C.); alfredo.conti2@unibo.it (C.A.); carlo.bortolotti@isnb.it (B.C.); 3 Department of Medical and Surgical Sciences (DIMEC), University of Bologna, 40126 Bologna, Italy; marta.linari@studio.unibo.it; 4 Department of Biomedical and Neuromotor Sciences—DIBINEM, University of Bologna, 40138 Bologna, Italy; matteo.zoli4@unibo.it (Z.M.); diego.mazzatenta@unibo.it (M.D.);; 5 IRCCS Istituto delle Scienze Neurologiche di Bologna, Programma Neurochirurgia Ipofisi—Pituitary Unit, 40126 Bologna, Italy; 6 IRCCS Istituto delle Scienze Neurologiche di Bologna, Epidemiology and Statistics Unit, 40139 Bologna, Italy; corrado.zenesini@isnb.it (Z.C.); lauramariabeatrice.belotti@ausl.bologna.it (B.L.M.B.); 7 IRCCS Istituto delle Scienze Neurologiche di Bologna, Anesthesia and Neurointensive Care Unit, 40126 Bologna, Italy; carloalberto.castioni@ausl.bologna.it; 8 IRCCS Istituto delle Scienze Neurologiche di Bologna, UOC Neuroradiologia, 40139 Bologna, Italy; c.princiotta@isnb.it (P.C.); m.dallolio@isnb.it (D.M.)

**Keywords:** aneurysmal subarachnoid hemorrhage, poor-grade, WFNS IV, WFNS V, cerebral aneurysm

## Abstract

Background: A subarachnoid hemorrhage due to an aneurysmal rupture (aSAH) is a serious condition with severe neurological consequences. The World Federation of Neurosurgical Societies (WFNS) classification is a reliable predictor of death and long-term disability in patients with aSAH. Poor-grade neurological conditions on admission in aSAH (PG-aSAH) are often linked to high mortality rates and unfavorable outcomes. However, more than one-third of patients with PG-aSAH may recover and have good functional outcomes if aggressive treatment is provided. We developed a risk model called Identifying POor GRade Outcomes (IPOGRO) to predict 6-month mRS outcomes in PG-aSAH patients as a secondary analysis of a previously published study. Methods: All consecutive patients in poor-grade neurological conditions (WFNS IV-V) admitted to our institute from 2010 to 2020 due to aSAH were considered. Clinical and neuroradiological parameters were employed in the univariable analysis to evaluate the relationship with a 6-month modified Rankin Scale (mRS). Then, a multivariable multinomial regression model was performed to predict 6-month outcomes. Results: 149 patients with PG-aSAH were included. Most patients were surgically treated, with only 33.6% being endovascularly treated. The 6-month mRS score was significantly associated with clinical parameters on admission, such as lowered Glasgow Coma Scale (GCS), leukocytosis, hyperglycemia, raised Systolic Blood Pressure (SBP), greater Simplified Acute Physiology Score (SAPS II score), increased initial serum Lactic Acid (LA) levels, and the need for Norepinephrine (NE) administration. Neuroradiological parameters on the initial CT scan showed a significant association with a worsening 6-month mRS. The IPOGRO risk model analysis showed an association between a WFNS V on admission and a poor outcome (mRS 4-5), while raised SBP was associated with mortality. Conclusions: Our IPOGRO risk model indicates that PG-aSAH patients with higher SBP at admission had an increased risk of death at 6-month follow-up, whereas patients with WFNS grade V at admission had an increased risk of poor outcome but not mortality.

## 1. Introduction

A subarachnoid hemorrhage caused by an aneurysmal rupture (aSAH) is a serious condition that can have severe neurological consequences. Despite reports of a recent decrease in mortality, SAH survivors frequently suffer from severe disabilities, cognitive deficits, and mental health issues [1,2].

The World Federation of Neurosurgical Societies (WFNS) classification [3] for the neurological condition at admission [4,5] is one of the most accurate predictors of death and long-term disability in patients with aSAH. However, several studies have found that high mortality rates and poor functional outcomes at long-term follow-up (FU) are frequently associated with poor-grade aSAH (PG-aSAH), which is defined as WFNS grades IV and V [6].

Nonetheless, aggressive treatment may allow approximately one-third of patients with aSAH admitted in poor neurological conditions (WFNS IV-V) to recover and have a good functional outcome at long-term FU [7]. As a result, PG-aSAH patients should not have their life support withdrawn solely because of their neurological grade at the time of admission. Despite efforts to predict long-term outcomes in PG-aSAH, most previous studies included patients with broad admission neurological grades, excluded those who could not be treated, and analyzed these patients without detailed stratification [8,9,10,11].

To specifically predict long-term outcomes in patients with PG-aSAH, we created a risk model called Identifying POor GRade Outcomes (IPOGRO), which considers neurological and systemic conditions, admission laboratory exam results, and neuroradiological features.

## 2. Materials and Methods

We followed the STROBE (Strengthening the Reporting of Observational Studies in Epidemiology) recommendations [12] and used a historical cohort design. This is a secondary analysis of a previously published article [13], whose primary goal was to investigate the factors that affect 30-day mortality in PG-aSAH. Using our prospective registry, we retrospectively evaluated all consecutive patients admitted in poor neurological condition (WFNS IV and V) to the IRCCS Istituto delle Scienze Neurologiche di Bologna due to aSAH between January 2010 and December 2020. All aSAH patients, including those who died before receiving aneurysm treatment, with a confirmed aneurysmal cause and a full clinical evaluation at admission, were included. We specifically included all patients who had an admission diagnosis of PG-aSAH, a full clinical evaluation, at least one CT scan examination, and one pre-operative neuroradiological examination for the aneurysmal source of bleeding (we considered either CT angiography or digital subtraction angiography—DSA). Our study had no age or gender limitations, our institution’s ethics committees approved the research, and we followed the *Helsinki Declaration*. However, due to the retrospective nature of the study, informed consent was waived.

### 2.1. Data Evaluations

Patients’ charts were reviewed to obtain demographic information, clinical evaluations, laboratory results, and medical treatment. The baseline demographics were age and gender, while clinical evaluations included neurological signs (such as seizure, anisocoria, mydriasis, miosis, and non-reacting pupils) and severity scores at admission (WFNS and Glasgow Coma Scale—GCS).

The admission laboratory examination included hematocrit (%), hemoglobin (g/dL), red blood cell (RBC) count (10^12^/L), presence/absence of leukocytosis (white blood cell—WBC ≥ 15 × 10^9^/L) [11], platelet count (10^9^/L), presence/absence of hyperglycemia (≥180 mg/dL), and activated partial thromboplastin time (aPTT). We also considered the mean value of the mean arterial pressure (MAP) on admission (mmHg), the presence/absence of invasively measured raised systolic blood pressure (SBP ≥ 180 mmHg), the presence/absence of acute onset of hypoxemia (arterial partial pressure of oxygen to fraction of inspired oxygen [PaO2/FIO2] ≤ 200 mmHg) [14], the Simplified Acute Physiology Score II (SAPS II) on admission, the presence/absence of increased values of procalcitonin (PCT ≥ 0.1 ng/mL), the presence/absence of increased initial serum lactic acid levels (LA ≥ 1.5 mMol/L), and the need for Norepinephrine (NE) administration on admission.

**Figure 1:**
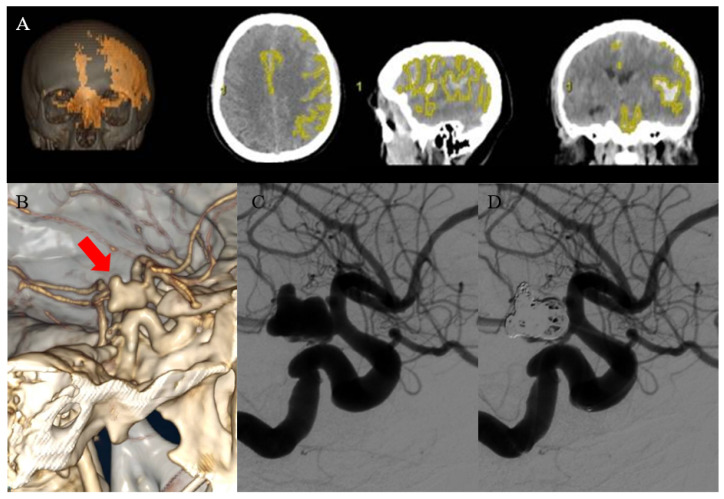
A case of a 63-year-old woman admitted with PG-aSAH (WFNS IV) due to the rupture of a posterior communicating artery aneurysm who presented a 6-month mRS of 2. (**A**) The CT scan on admission with semiautomatic hemorrhage volume evaluation in volume rendering (VR), axial, sagittal and coronal. (**B**) CT angiography demonstrating the presence of the ruptured posterior communicating artery aneurysm (arrow). DSA of the aneurysm before (**C**) and after (**D**) the endovascular treatment.

All admission CT scans were reviewed by an interventional neuroradiologist and a vascular neurosurgeon with over seven and ten years of experience, respectively. They were blinded to the patients’ clinical characteristics, outcomes, and each other’s work with the aim to extract data such as the modified Fisher grade (mFISHER), the number and presence/absence of blood into brain ventricles, the measure of median line shift (mm), the presence/absence of acute hydrocephalus, the presence/absence of subdural hemorrhage, the presence/absence of cerebral herniation, and the presence/absence of rebleeding. Aneurysm rebleeding was considered in the event of sudden clinical deterioration accompanied by increased subarachnoid, intracerebral, or ventricular blood on a subsequent CT scan. Admission CT scans were then used to calculate the amount of intracranial bleeding caused by cerebral aneurysmal rupture using Advantage Workstation (AW) Server 3.2, a software-based semi-automatic segmentation tool from GE Healthcare (Chicago, IL, USA). To avoid bone-related artifacts, the intracranial blood volume was first automatically assessed using the Stroke Volume Computer Assisted Reading (VCAR) tool on the admission non-contrast CT scan, then manually adjusted, reviewed, and confirmed. Each case underwent manual threshold-based correction because blood mixed with cerebrospinal fluid (CSF) appears more hypodense, resulting in incorrect automatic segmentations [15]. The CT image analysis software package automatically calculated all volumes in mm^3^ (Figure 1).

The 6-month outcome for mRS was obtained from the patient’s charts and FU. It was classified into three categories: the first (good outcome) with an mRS score of 1 to 3, the second (poor outcome) with an mRS score of 4 to 5, and the third (patient death) with an mRS score of 6.

### 2.2. Statistical Analysis

Categorical variables are presented as absolute numbers (*n*) and percentages (%), while continuous variables are presented as mean and standard deviation (SD) or median and interquartile range (IQR), as appropriate. Variable differences were compared among patients with good outcomes (mRS 1-2-3), poor outcomes (4-5) and those who died (mRS 6) at 6-month FU, including both diagnostic laboratory parameters, clinical evaluations and neuroradiological examinations on admission. The differences between the three groups were assessed by the Kruskal–Wallis rank test for continuous variables and by Pearson chi-square test for categorical ones. Associations among clinical and neuroradiological features and the 6-month outcome were assessed by the Pearson chi-square test and univariable multinomial regression models (with mRS as the dependent variable and good outcome as the reference category). Then, variables associated with the univariable analyses were included in a multivariable multinomial regression model. Results are presented as Relative Risk Ratios (RRR) with 95% Confidence Interval (95% CI). A sub-analysis on patients with WFNS IV on admission was also performed. For the statistical analysis, a *p*-value < 0.05 was considered significant; all statistical analyses were performed using Stata SE version 14.2.

## 3. Results

A total of 149 PG-aSAH patients were included in this study. Of them, 108 (72.5%) patients were female, with a mean age of 61.3 ± 11.9 years (range: 29–86 years). The 66.4% of patients presenting with PG-aSAH were treated surgically with clipping, while 33.6% were treated endovascularly by coiling. In our series, the 30-day mortality was 20.8%, and only eight patients (7% of the remaining population) died later during hospitalization. Clinical and neuroradiological parameters considered in this study are shown in Table 1 and Table 2.

**Figure 2:**
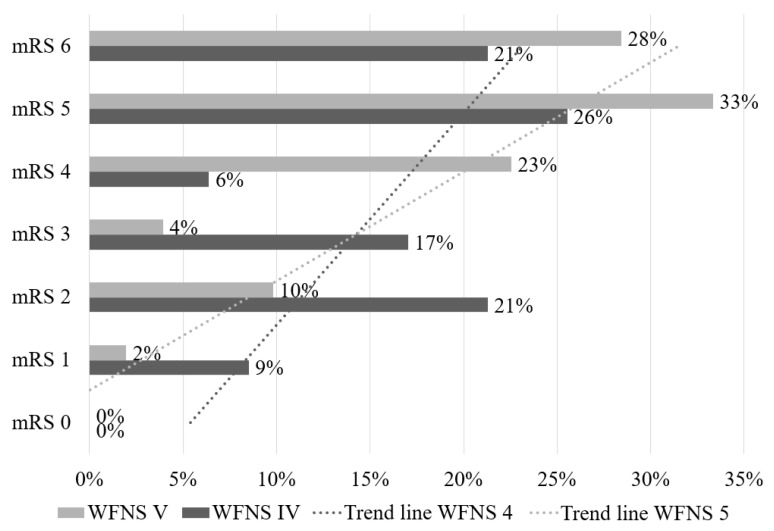
The 6-month mRS for our poor-grade aneurysmal SAH series. Patients admitted with WFNS IV had a significantly better outcome than those admitted with WFNS V, with trend lines rising in tandem with a clear overall data slope and crossing at mRS 3.

Admission 47 (31.5%) presented a WFNS IV, while 102 (68.5%) presented a WFNS V. Among the patients presented with WFNS IV on admission, the 6-month mRS was 1 in 9%, 2 in 21%, 3 in 17%, 4 in 6%, 5 in 26%, and 6 in 21% of cases; for patients presented with WFNS V the 6-month mRS was 1 in 2%, 2 in 10%, 3 in 4%, 4 in 23%, 5 in 33%, and 6 in 28% of cases. None of our patients recovered completely with a mRS score of 0 at the 6-month FU (Figure 2).

We discovered a statistically significant association between a worsening in 6-month mRS outcomes and clinical parameters on admission, such as a lower GCS score (*p* = 0.001), a WFNS V (*p* = 0.002), the presence of leukocytosis (*p* = 0.012), hyperglycemia (*p* = 0.006), raised SBP (*p* = 0.004), and the need for NE administration on admission (*p* = 0.033) (Table 1). Among neuroradiological parameters on the initial CT scan, those who presented a significant statistical association with a worsening in the 6-month outcome were a mFISHER of 4 (*p* = 0.008), a greater amount of blood (*p* = 0.001), an increased number of ventricles with hemorrhage (*p* = 0.013), the presence of blood in both lateral ventricles (*p* = 0.001), and the presence of blood in the third ventricle (*p* = 0.010). Only the presence of rebleeding (*p* = 0.010) was found to have a significant statistical association with a worsening 6-month outcome in subsequent CT scan evaluations (Table 2). During hospitalization, a statistically significant difference in the 6-month outcomes and the need for decompressive craniotomy (*p* = 0.002), the need for VP shunt (*p* = 0.001), a higher SAPS II score (*p* = 0.001) and the presence of increased initial serum LA levels (*p* = 0.026) were demonstrated (Table 3).

The multivariable multinomial regression model (IPOGRO risk model analysis) showed an association between WFNS V on admission (*p* = 0.016) and a poor outcome (mRS 4-5) with an RRR of 3.47 (95% CI: 1.26–9.56) compared to a good outcome (mRS 1-2-3). Instead, the presence of raised SBP on admission (*p* = 0.004) was associated with mortality (mRS 6) compared to a good outcome (mRS 1-2-3) with a RRR of 6.99 (95% CI: 1.86–26.28). A greater total volume of blood and the presence of hyperglycemia demonstrated an association, without statistical significance (respectively *p* = 0.068; and *p* = 0.097) with mortality (mRS 6) compared to a good outcome (mRS 1-2-3) at 6-month FU, with RRR of 1.04 (95% CI: 0.99–1.09), and of 2.75 (95% CI: 0.83–9.06), respectively (Table 4).

The sub-analysis on patients with WFNS IV on admission (Table 5) demonstrated the association between raised SBP on admission (*p* = 0.020) and mortality (mRS 6) with a RRR of 14.16 (95% CI: 1.51–132.72) compared to a good outcome (mRS 1-2-3) at 6-month FU.

## 4. Discussion

In this study, we reported our single center experience with aggressive treatment of PG-aSAH patients (WFNS IV and V) to identify those who may recover at long term FU. As previously reported in other series, even in our study the mortality of PG-aSAH remains high (26.2% overall, with 20.8% mortality in the first month) [5,16,17], especially in patients with recurrent bleeding [18].

However, 25.5% of patients admitted in poor neurological condition (WFNS IV and V) had a 6-month good outcome (mRS 1-2-3), with higher percentages in the group of patients with WFNS IV on admission compared to WFNS V, indicating that there may be other factors involved in better outcomes besides neurological status at admission [19,20,21]. In contrast, the percentages of poor outcome (mRS 4-5) and death (mRS 6) were similar in patients with both WFNS IV and V on admission, and none of PG-aSAH patients in our series recovered completely with a mRS 0 at 6-month FU, thus reiterating the concept that PG-aSAH is a serious disease (Figure 2).

Based on these considerations, we sought to identify PG-aSAH patients who would benefit from aggressive treatment, given their young mean age (61.3 years), as at least one-third of them can achieve independence and resume working, rather than selecting patients for withdrawal of life support based solely on neurological status at admission [5,22,23,24,25,26].

In our IPOGRO risk model, patients with a WFNS V at admission had a statistically significant (*p* = 0.016) higher risk of poor outcome (mRS 4-5) at 6-month FU than others, with an RRR of 3.47 when compared to a good 6-month outcome. Elevated SBP at admission is a statistically significant predictor of mortality (*p* = 0.004), with a high RRR of 6.99 when compared to a favorable 6-month outcome (mRS 1-2-3). Although not statistically significant, there was an association between a GCS of 3 on admission (*p* = 0.086), hyperglycemia (*p* = 0.097), and an increased volume of blood on CT scan (*p* = 0.068) and mortality, with death patients having a higher RRR than those who had a good 6-month outcome (mRS 1-2-3).

Because the group of patients with WFNS IV on admission had a higher percentage of 6-month good outcome (mRS 1-2-3) (Figure 2), we decided to conduct a sub-analysis to see if other factors contributed to the higher chance of 6-month good outcome compared to patients with WFNS V on admission. A higher SBP at admission was associated with a statistically significant (*p* = 0.020) increased risk of death, with a very high RRR of 14.16 when compared to a good 6-month outcome (mRS 1-2-3). There were no other factors linked to an increased risk of poor outcome (mRS 4-5) or mortality (mRS = 6) at 6-month FU (Table 5).

As a result, regardless of WFNS grade at admission, our IPOGRO risk model found a significant statistical association with 6-month mortality for patients who had elevated SBP at admission. Unfortunately, no other parameters were found to be associated with an increased risk of a poor outcome at 6-month follow-up.

While all patients with PG-aSAH should be treated aggressively, those with elevated SBP on admission, both WFNS IV and V, should be considered for more intensive care, as well as providing information to family members while in the hospital, especially if their condition worsens. In fact, approximately 25% of patients admitted with poor neurological conditions (WFNS IV and V) have a good 6-month outcome (mRS 1-2-3), and they are usually young [22,23,24,25,26].

Some of our study’s main limitations include the small sample size, a single center experience, and the inability to assess every variable that could be involved in a complex pathology like aSAH which can necessitate a lengthy recovery period in an intensive care unit, and the onset of various neurological and/or systemic complications. To give patients with PG-aSAH the best chance of recovery, we hope that future research will be able to identify which factors are involved in worsening and require aggressive treatment.

## 5. Conclusions

Our IPOGRO risk model revealed that PG-aSAH patients with higher SBP at admission had a higher risk of death at 6-month follow-up, indicating that blood pressure control is still critical for reducing the risk of rebleeding and preventing ischemic complications by avoiding a sudden drop in blood pressure.

Interestingly, in our model, patients with WFNS grade V at admission had an increased risk of poor outcome but not mortality. More research is needed to determine which factors contribute to the worsening of PG-aSAH, and which patients require more intensive care to increase the likelihood of a positive outcome.

## Figures and Tables

**Table 1:** Clinical parameters on admission by 6-month mRS category.

	mRS 1-2-3	mRS 4-5	mRS 6	*p*-Value
Age [years]	58.7 ± 12.6	63.1 ± 10.8	60.2 ± 10.8	0.246
Female sex	26 (72.2%)	54 (73.0%)	28 (71.8%)	0.990
Seizures	5 (13.9%)	6 (8.1%)	0	>0.999
Mean GCS score	7.0 ± 2.9	5.3 ± 2.5	4.6 ± 2.1	**0.001**
WFNS V	16 (44.4%)	57 (77.0%)	29 (74.4%)	**0.002**
Anticoagulant therapy	0	2 (2.6%)	1 (2.5%)	0.613
Antiplatelet therapy	4 (11.1%)	16 (21.6%)	8 (20.5%)	0.395
Anisocoria	5 (13.9%)	22 (29.7%)	6 (15.4%)	*0.085*
Mydriasis	1 (2.8%)	8 (10.8%)	5 (12.8%)	0.278
Miosis	9 (25.0%)	19 (25.7%)	17 (43.6%)	0.106
Hematocrit [%]	0.9 ± 0.3	1.5 ± 4.8	1.9 ± 5.5	0.772
Hemoglobin [g/dL]	13.6 ± 1.8	13.2 ± 1.8	13.4 ± 1.9	0.702
RBC count [10^12^/L]	4.6 ± 0.7	4.5 ± 0.7	4.6 ± 0.7	0.992
Leukocytosis [WBC ≥ 15 × 10^9^/L]	11 (30.6%)	25 (33.8%)	20 (52.6%)	*0.088*
WBC count [10^9^/L]	12.9 ± 4.4	13.6 ± 4.8	16.9 ± 6.4	**0.012**
Platelet count [10^9^/L]	260.4 ± 72.7	243.5 ± 78.7	273.3 ± 93.9	0.173
Hyperglycemia [≥180 mg/dL]	7 (19.4%)	29 (39.7%)	23 (59.0%)	**0.002**
aPTT ratio	0.9 ± 0.3	1.5 ± 4.8	1.9 ± 5.5	0.555
SBP ≥ 180 mmHg	5 (13.9%)	21 (28.4%)	19 (48.7%)	**0.004**
NE administration	15 (41.7%)	45 (60.8%)	27 (71.1%)	**0.033**

GCS = Glasgow Coma Scale; WFNS = World federation of neurosurgical societies; RBC = Red blood cells; WBC = White blood cells; aPTT = activated partial thromboplastin time; SBP = Systolic blood pressure; NE = Norepinephrine. Statistically significant *p*-value are highlighted in bold; near statistically significant *p*-value are highlighted in italics.

**Table 2:** Neuroradiological parameters on admission by 6-month mRS category.

	mRS 1-2-3	mRS 4-5	mRS 6	*p*-Value
m-FISHER 4 on CT	25 (69.4%)	64 (86.5%)	37 (94.9%)	**0.008**
N. of ventricles with blood	2.2 ± 1.7	3.0 ± 1.5	3.3 ± 1.3	**0.013**
Intraventricular hemorrhage	25 (69.4%)	64 (86.5%)	37 (94.9%)	**0.008**
Hemorrhage in lateral ventricles	21 (58.3%)	62 (83.8%)	35 (89.7%)	**0.001**
Hemorrhage in the 3rd ventricle	18 (50.0%)	55 (74.3%)	31 (79.5%)	**0.010**
Hemorrhage in the 4th ventricle	22 (61.1%)	54 (73.0%)	31 (79.5%)	0.200
Intraventricular blood volume [mm^3^]	18.9 ± 26.4	20.8 ± 26.9	19.2 ± 25.8	**0.001**
Hydrocephalus on admission	27 (75.0%)	54 (73.0%)	30 (76.9%)	0.898
Intraparenchimal hemorrhage	23 (63.9%)	55 (74.3%)	29 (74.4%)	0.479
Intraparenchimal blood volume [mm^3^]	25.7 ± 23.3	18.2 ± 23.8	12.6 ± 22.4	**0.021**
Total volume of blood [mm^3^]	5.7 ± 9.3	18.4 ± 26.2	26.1 ± 27.6	**0.001**
Median line shift [mm]	2.6 ± 3.0	5.1 ± 5.3	3.7 ± 3.7	0.115
Subdural hemorrhage	6 (16.7%)	18 (24.3%)	5 (12.8%)	0.302
Cerebral herniation	3 (8.3%)	14 (18.9%)	6 (15.4%)	0.354
Vertebro-basilar aneurysm	4 (11.1%)	9 (12.2%)	10 (25.6%)	0.120
Spot sign	0	3 (4.1%)	3 (7.7%)	0.329
Vessel involvement in aneurysmal neck	11 (30.6%)	16 (21.6%)	8 (20.5%)	0.513
Aneurysm’ re-bleeding	1 (2.8%)	7 (9.5%)	8 (20.5%)	**0.041**
m-FISHER 4 on CT	25 (69.4%)	64 (86.5%)	37 (94.9%)	**0.008**

Statistically significant *p*-value are highlighted in bold.

**Table 3:** Clinical and radiological parameters during hospitalization by 6-month mRS category.

	mRS 1-2-3	mRS 4-5	mRS 6	*p*-Value
Decompressive craniotomy	2 (5.6%)	25 (33.8%)	15 (38.5%)	**0.002**
DCI	12 (33.3%)	29 (39.2%)	18 (46.2)	0.523
VP shunt	13 (36.1%)	35 (47.3%)	3 (7.9%)	**0.001**
PaO2/FIO2 ≤ 200 mmHg	6 (16.7%)	14 (18.9%)	12 (30.8%)	0.249
SAPS II score	38.5 ± 12.7	50.0 ± 12.2	52.1 ± 12.8	**0.001**
PCT ≥ 0.1 ng/mL	18 (50.0%)	48 (64.9%)	18 (47.4%)	0.134
LA ≥ 1.5 mMol/L	27 (75.0%)	61 (82.4%)	36 (97.3%)	**0.026**

DCI = Delayed Cerebral Ischemia; VP = Ventriculoperitoneal; PaO2 = Arterial partial pressure of oxygen; FIO2 = Fraction of inspired oxygen; SAPS = Simplified acute physiology score; PCT = Procalcitonin; LA = Lactic acid. Statistically significant *p*-value are highlighted in bold.

**Table 4:** Multivariable multinomial regression model (IPOGRO risk model analysis) with mRS score category as the dependent variable.

		RRR	95% CI	*p*-Value
mRS 1-2-3	(reference)			
mRS 4-5	GCS score = 3	0.87	0.25–2.95	0.820
	WFNS score = V	3.47	1.26–9.56	**0.016**
	SBP ≥ 180 mmHg	2.68	0.80–8.92	0.109
	Hyperglycemia [≥180 mg/dL]	1.79	0.63–5.07	0.276
	N. of ventricles with blood	1.16	0.85–1.59	0.341
	Total volume of blood [mm^3^]	1.03	0.99–1.08	0.119
mRS 6	GCS score = 3	3.64	0.83–15.98	*0.086*
	WFNS score = V	1.08	0.27–4.23	0.915
	SBP ≥ 180 mmHg	6.99	1.86–26.28	**0.004**
	Hyperglycemia [≥180 mg/dL]	2.75	0.83–9.06	*0.097*
	N. of ventricles with blood	1.41	0.93–2.11	0.102
	Total volume of blood [mm^3^]	1.04	0.99–1.09	*0.068*

RRR = Relative Risk Ratio; 95% CI = 95% Confidence Interval. Statistically significant *p*-value are highlighted in bold; near statistically significant *p*-value are highlighted in italics.

**Table 5:** Multivariable multinomial regression model with mRS score category as dependent variable on patients with WFNS IV on admission.

		RRR	95% CI	*p*-Value
mRS 1-2-3	(reference)			
mRS 4-5	SBP ≥ 180 mmHg	4.57	0.53–39.40	0.166
	Hyperglycemia [≥180 mg/dL]	1.95	0.51–7.51	0.329
	N. of ventricles with blood	1.09	0.70–1.69	0.691
	Total volume of blood [mm^3^]	1.03	0.98–1.08	0.253
mRS 6	SBP ≥ 180 mmHg	14.16	1.51–132.72	**0.020**
	Hyperglycemia [≥180 mg/dL]	2.12	0.46–9.81	0.334
	N. of ventricles with blood	1.14	0.66–1.94	0.642
	Total volume of blood [mm^3^]	1.03	0.98–1.09	0.243

RRR = Relative Risk Ratio; 95% CI = 95% Confidence Interval. Statistically significant *p*-value are highlighted in bold.

## Data Availability

The raw data supporting the conclusions of this article will be made available by the authors on request.
